# Health-Related Quality of Life in Children With Kaposiform Hemangioendothelioma

**DOI:** 10.3389/fped.2022.720611

**Published:** 2022-02-24

**Authors:** Shiyi Dai, Kaiying Yang, Tong Qiu, Jiangyuan Zhou, Xuepeng Zhang, Siyuan Chen, Lizhi Li, Yi Ji

**Affiliations:** ^1^Division of Oncology, Department of Pediatric Surgery, West China Hospital of Sichuan University, Chengdu, China; ^2^Pediatric Intensive Care Unit, Department of Critical Care Medicine, West China Hospital of Sichuan University, Chengdu, China; ^3^Department of Pediatric Surgery, Shengli Clinical Medical College of Fujian Medical University, Fuzhou, China

**Keywords:** kaposiform hemangioendothelioma, Kasabach-Merritt phenomenon, quality of life, PedsQL™ 4.0, vascular tumor

## Abstract

**Background and Objective:**

Kaposiform hemangioendothelioma (KHE) is a rare, aggressive and borderline vascular tumor mainly occurring in infants and children. The aim of this study was to determine the health-related quality of life (HRQOL) in children with KHE.

**Measures:**

A total of 91 children with KHE participated in this cross-sectional study. The HRQOL was assessed by the age-specific Pediatric Quality of Life Inventory Version 4.0 (PedsQL™ 4.0) Infant Scales, Family Information Form (FIF), Family Impact Module (FIM) and Generic Core Scales (GCS). For comparison, demographically matched healthy children were recruited as a control group. The main outcome measure of HRQOL was analyzed in the two groups. We determined related factors that influenced the HRQOL in children with KHE and their parents by using a stepwise multiple regression analysis.

**Results:**

The study found that the scores of each item in the family impact module (FIM) were lower than 75, which suggesting that KHE can make the parents of patients in a state of poor quality of life. The scores of physiological and psychosocial domains in all age groups of patients with KHE were lower than those of normal children (*P* < 0.01). Activity dysfunction is the factor influencing the physiological function score of all-age patients. KMP is the factor influencing the psychosocial function score of all-age patients.

**Conclusions:**

The findings presented here suggest that patients with KHE have a poor HRQOL. KMP and activity dysfunction are risk factors for poor HRQOL in patients with KHE. However, lesion size, lesion location and education level of the mother and father were not related to the HRQOL.

## Introduction

Kaposiform hemangioendothelioma (KHE) is a rare, endodermic, aggressive and borderline vascular tumor mainly occurring in infants and children (1). The incidence rate of KHE is 0.0091‰ (2). Approximately 70% of KHE cases are associated with thrombocytopenia, coagulation factor depletion and severe anemia, known as the Kasabach-Merritt phenomenon (KMP) (3). In recent years, multiple lesions and complex cases of KHE have been increasingly reported (4–8). However, there is currently no appropriate assessment of quality of life (QOL) in patients with KHE. It is still unknown whether KHE affects the QOL in children with KHE and their parents.

Health-related QOL (HRQOL) is a psychosocial response to a patient's disease and treatment. The HRQOL is affected by the disease itself, treatment and long-term complications. Quantitative assessments of QOL have resulted in substantial changes in health measurements. We know that the QOL in normal children is related to family economic status, the intimate relationship between family members, level of socioeconomic culture development and the behavior concept of children's education. We suspect that the frequency and severity of disease onset, negative emotions such as anxiety and depression, an insufficient understanding of the disease by parents or other factors may have an effect and are worth exploring (9, 10). For evaluating HRQOL in patients with KHE, we know that the progression of the KHE disease is often accompanied by life-threatening KMP and musculoskeletal complications. KHE can cause damage to children's joint function, resulting in long treatment and recovery time. Some cases of KHE are disabled and deformed. The influence of the lesion itself, the sequelae of surgical treatment, and the side effects of long-term use of hormones, vincristine, sirolimus and other drugs may affect the normal life of children. Therefore, we need to pay special attention to the QOL of in children with KHE, and take corresponding measures to improve their QOL and avoid psychological and social behavior problems in their growth stage.

In the present study, we used the objective Pediatric QOL Inventory Version 4.0 (PedsQL™ 4.0) scale to evaluate patients with KHE, explore the impact of the disease on patients and understand the main factors affecting patients' HRQOL. In this way, we assessed the HRQOL of KHE children and their parents as well as the main influencing factors and developed targeted intervention measures to improve their HRQOL.

## Methods

### Participants

The research was conducted in the Pediatric Surgery Department, West China Hospital of Sichuan University and the Department of Children Preventive Health Care, West China Second Hospital. Both hospitals are tertiary medical centers that serve children referred by pediatricians and surgeons. This study was approved by the ethics committees of the West China Hospital of Sichuan University and West China Second University Hospital of Sichuan University. All procedures followed approved research protocols. We recruited 0–14-year-old children diagnosed with KHE at the Department of Pediatric Surgery, West China Hospital of Sichuan University, and coetaneous healthy children at the Department of Children Preventive Health Care, All the recruited patients were newly diagnosed with KHE by a multidisciplinary vascular anomaly group (a collaboration team including IH experts in pediatric surgery, plastic surgery, pediatric dermatology and radiology) at our hospital. West China Second Hospital, from January 2018 to July 2020. The parents of the children enrolled in the study signed informed consent forms. All questionnaires were completed and received at the time of enrollment.

### Instruments

All children and parents were administered a questionnaire to assess the HRQOL. The PedsQL™ 4.0 Chinese versions, which contain the Family Information Form (FIF), Family Impact Module (FIM), Generic Core Scales (GCS) and Infant Scales, were assessed. The PedsQL™ 4.0 is a reliable and validated multidimensional modular approach to measuring HRQOL in children and adolescents (11–14). The PedsQL™ 4.0 GCS includes physiological and psychosocial domains which consists of 4 functional domains, including physical, emotional, social, and school functioning, and 4 different ages groups: 2–4 years (21 items), 5–7 years (23 items), 8–12 years (23 items), and 13–18 years (21 items). The PedsQL™ 4.0 Infant Scales were used for children aged 1–12 and 13–24 months. The 1–12 month scale contains 38 items, and the 13–24 month scale contains 45 items. Both cover 5 functional domains: physical, physical symptom, emotional, social and cognitive functioning. Each item is a question on the frequency of something happening in the last month. KHE children and their parents were required to complete the PedsQL™ 4.0 GCS or PedsQL™ 4.0 Infant Scales, PedsQL™ 4.0 FIF and PedsQL™ 4.0 FIM. Healthy children and their parents needed to complete only the PedsQL™ 4.0 GCS or PedsQL™ 4.0 Infant Scales and PedsQL™ 4.0 FIF. The questionnaires for the age groups 1–12 months, 13–24 months, and 2–4 years were answered by parents, whereas the questionnaires for the age groups 5–7, 8–12, and 13–18 years were answered by both the children themselves and their parents. All questionnaires used a Likert-type scale, where 0 was never, 1 was almost never, 2 was sometimes, 3 was often and 4 was almost always. Scores of 0–4 for each item were converted to a 0–100 scale (0 = 100, 1 = 75, 2 = 50, 3 = 25, 4 = 0). Scores for each item ranged from 0 to 100, and high scores indicated a good HRQOL. Furthermore, questionnaires with incomplete basic information and more than half of items missing were considered invalid.

### Procedures

Before completing the questionnaire, we obtained informed consent from the parents and provided a good explanation of the purpose and significance of the research. Under the supervision of trained physicians, when necessary, the staff explained the study to the parents individually. The children themselves or their parents filled in the general information and questionnaire items. Children who were older than 5 years of age completed the PedsQL™ 4.0 scale module independently. In addition, the clinical data of patients with KHE were reviewed after verification by 2 investigators.

### Statistical Analysis

The general characteristics of the patients with KHE are presented using descriptive statistics. For quantitative data with a normal distribution, the mean ± standard deviation (SD) is presented. Independent sample *t*-tests were used for comparisons of differences between groups. For quantitative data with a non-normal distribution, medians with interquartile intervals are presented. For categorical data, comparisons between groups were performed using chi-square (χ^2^) tests and multiple regression analysis. *P* < 0.05 was considered to be statistically significant. All statistical analyses were performed with SPSS 24.0 statistical software (SPSS Inc., Chicago, USA).

## Results

In total, 182 questionnaires were distributed, and 177 questionnaires were effectively recovered, with a recovery rate of 97.25%. A total of 177 children participated in the study, including 91 patients with KHE (54 male, 37 female) and 86 healthy children (47 male, 39 female). The mean age of the patients was 35.82 ± 41.27 months, media age was 18 months, range from 1 months to 165 months (IQR 5-59). The mean age of the healthy children was 36.10 ± 41.63 months, media age was 22.5 months, range from 1 months to 157 months (IQR 11-40.75). According to the applicable scales, we divided the participants into two age groups (age ≤24 months and age >24 months). Table 1 shows the baseline characteristics of the children with KHE and those in the normal group. Age, gender, the relationship between the respondents, and mother's or father's education level were not significantly different between the KHE group and the control group.

**Table 1:** Baseline characteristics of the children in the KHE and normal groups.

**Variables**	**KHE**	**Normal**	***P*-values**
	***n* = 91**	***n* = 86**	
Age^†^	35.82 ± 41.27	36.10 ± 41.63	0.344
Age ≤24 months	53 (58.24%)	43 (50.00%)	
Age >24 mouths	38 (41.76%)	43 (50.00%)	
Gender^‡^			0.527
Male	54 (59.34%)	47 (54.65%)	
Female	37 (40.66%)	39 (45.35%)	
Relationship between the respondents^‡^			0.568
Father	25 (27.47%)	18 (20.93%)	
Mother	52 (57.14%)	52 (60.47%)	
Father and mother	14 (15.39%)	16 (18.60%)	
Mother's education level^‡^			0.928
Elementary education	19 (20.88%)	16 (18.60%)	
Secondary education	18 (19.78%)	18 (20.93%)	
Higher education	54 (59.34%)	52 (60.47%)	
Father's education level^‡^			0.664
Elementary education	21 (23.08%)	18 (20.93%)	
Secondary education	19 (20.88%)	14 (16.28%)	
Higher education	51 (56.04%)	54 (62.79%)	

*KHE, Kaposiform hemangioendotheliomas; KMP, Kasabach-Merritt phenomenon*.

† *Data are shown as the mean and standard deviation*.

‡ *Values are presented as the number (percentage)*.

The demographic details and clinical data of the KHE group are listed in Table 2. Among the 91 study subjects, the average tumor diameter was 7.91 ± 4.32 cm. 48 (52.75%) patients had KMP, and 43 (47.25%) patients did not. The tumors were located on the head, face or neck in 23 (25.27%) cases, trunk in 32 (35.16%) cases, and extremities in 36 (39.56%) cases. Activity dysfunction in children with KHE was reported in 32 (35.16%) cases, and 59 (64.84%) cases were not associated with activity dysfunction. Some of the patients developed complications, including decreased range of motion, severe pain, coagulation disorders, active organ bleeding, etc.

**Table 2:** Demographic details and clinical data of the KHE group (*n* = 91).

**Variable**	**No. (% of total)**
Tumor size (average diameter in cm)	7.91 ± 4.32
**KMP**
With KMP	48 (52.75%)
Without KMP	43 (47.25%)
**Activity dysfunction**
With activity dysfunction	32 (35.16%)
Without activity dysfunction	59 (64.84%)
**Location**
Head face and neck	23 (25.27%)
Trunk	32 (35.16%)
Extremity	36 (39.56%)
**Complications**
Decreased range of motion	32 (35.16%)
Severe pain	9 (9.89%)
Coagulation disorders	48 (52.75%)
Active organ bleeding	4 (4.40%)
Acute heart failure	3 (3.30%)
Pleural effusion	2 (2.19%)
Pericardial effusion	5 (5.49%)

*KHE, Kaposiform hemangioendotheliomas; KMP, Kasabach-Merritt phenomenon*.

FIM showed that the scores of family members of patients with KHE in physiology, emotion, society, cognition, communication, worry, daily activities, family relationship and family financial burden were lower than 75, indicating that the parents of patients were in poor quality of life and KHE can reduce the quality of life of patients' guardians. We found that the scores of physiological and psychosocial function domains of patients with KHE were lower than those of normal children (all *P* < 0.01). The scores of HRQOL physiological and psychosocial function of the >24 months group were lower than the ≤24 months group.

Through the analysis of the influencing factors of HRQOL in patients with KHE, we found that the activity dysfunction was the factor influencing the score of physiological function, and KMP was the factor influencing the score of psychosocial function. In practical terms, The scores of each instrument in the patient group and healthy group are shown in Tables 3–5. There were significant differences in physical functioning, physical symptom functioning and emotional functioning in the PedsQL™ 4.0 Infant Scales (*P* < 0.05) scores between the ≤24 months group and the healthy ≤24 months group. However, no significant difference was revealed in social functioning and cognitive functioning (*P* > 0.05). Interestingly, when we used the PedsQL™ 4.0 GCS to compare the patients age >24 months with the healthy children aged >24 months, we found that physical, emotional, social and cognitive functioning were significantly different (*P* < 0.05).

**Table 3:** Scores of each instrument in the patient group and healthy group.

**Variables**	**Total patient group**	**≤24 months group**	**>24 months group**	**Healthy children aged ≤24 months**	**Healthy children age >24 months**	***P*-values^†^**
**PedsQL™ 4.0 family impact module**						
Physical functioning	63.92 ± 21.74	64.32 ± 22.68	63.36 ± 20.64	N/A	N/A	0.739
Emotional functioning	56.26 ± 27.21	58.21 ± 28.34	53.55 ± 26.68	N/A	N/A	0.199
Social functioning	61.33 ± 26.30	62.50 ± 27.60	59.70 ± 24.65	N/A	N/A	0.305
Cognitive functioning	63.88 ± 22.71	65.57 ± 25.32	61.54 ± 18.55	N/A	N/A	0.081
Communication	64.38 ± 23.03	65.38 ± 25.13	62.98 ± 19.97	N/A	N/A	0.237
Worry	43.79 ± 24.97	45.85 ± 25.98	40.92 ± 23.53	N/A	N/A	0.297
Daily activities	56.03 ± 28.73	55.16 ± 30.88	57.24 ± 25.79	N/A	N/A	0.893
Family relationships	73.46 ± 20.02	76.23 ± 21.26	69.61 ± 17.72	N/A	N/A	0.060
Financial issues	49.45 ± 30.95	50.94 ± 31.76	47.37 ± 30.08	N/A	N/A	0.541
**PedsQL**™ **4.0 infant scales**						
Physical functioning	N/A	75.26 ± 18.48	N/A	92.39 ± 9.49	N/A	0.000
Physical symptom functioning	N/A	80.94 ± 15.47	N/A	90.78 ± 7.84	N/A	0.002
Emotional functioning	N/A	69.74 ± 17.95	N/A	90.44 ± 8.17	N/A	0.000
Social functioning	N/A	87.78 ± 14.57	N/A	91.07 ± 10.69	N/A	0.089
Cognitive functioning	N/A	83.88 ± 19.49	N/A	88.12 ± 17.14	N/A	0.241
**PedsQL**™ **4.0 generic core scales**						
Physical functioning	N/A	N/A	65.87 ± 23.12	N/A	89.06 ± 8.23	0.000
Emotional functioning	N/A	N/A	70.00 ± 18.38	N/A	85.48 ± 13.47	0.000
Social functioning	N/A	N/A	75.39 ± 20.94	N/A	89.88 ± 14.42	0.002
Cognitive functioning	N/A	N/A	68.84 ± 20.93	N/A	91.38 ± 9.19	0.000

*Data are shown as the mean and standard deviation; N/A not available*.

† *For PedsQL™ 4.0 Family Impact Module, P-values represent patients aged ≤24 months compared with patients aged >24 months; For the PedsQL™ 4.0 Infant Scales and PedsQL™ 4.0 Generic Core Scales, P-values represent the patients groups compared with healthy children*.

**Table 4:** Scores of physiological and psychosocial functions in children with KHE and normal children.

	**Physiological function mean ±SD/95% CI**	**Psychosocial function mean ±SD/95% CI**
Total healthy children group	90.63 ± 8.29 (88.86–92.41)	89.99 ± 8.48 (88.17–91.81)
Total patient group	73.67 ± 19.08 (69.70–77.65)	77.73 ± 15.76 (74.45–81.01)
*P*-values	*P* < 0.01	*P* < 0.01
**PedsQL**™ **4.0 infant scales**		
≤24 months healthy children group	91.88 ± 8.20 (89.35–94.40)	89.74 ± 8.91 (87.01–92.48)
≤24 months patient group	78.10 ± 14.67 (74.06–82.14)	80.47 ± 14.66 (76.43–84.51)
*P*-values	*P* < 0.01	*P* < 0.01
**PedsQL**™**4.0 generic core scales**		
>24 months healthy children group	89.39 ± 8.28 (86.84–91.94)	90.24 ± 8.12 (87.74–92.74)
>24 months patient group	67.50 ± 22.71 (60.04–74.97)	73.92 ± 16.64 (68.45–79.39)
*P*-values	*P* < 0.01	*P* < 0.01

*Mean ± SD, mean and standard deviation; CI, Confidence Interval*.

**Table 5:** Multiple regression analysis of factors influencing quality of life score of patients with KHE.

	**Coeff (B)**	**Std Coeff (β)**	**Significance (*P*-value)**	**95% CI**
**Physiological function**				
KMP	0.006	0.194	0.065	0.960(0.913–1.011)
Activity dysfunction	0.005	0.003	0.048	1.071 (1.020–1.125)
Location	−0.007	−0.293	0.059	1.014 (0.963–1.068)
Mother's education level	0.006	0.140	0.183	0.957 (0.890–1.029)
Father's education level	0.008	0.191	0.069	1.019 (0.941–1.105)
**Psychosocial function**				
KMP	0.007	0.266	0.011	1.125 (1.052–1.203)
Activity dysfunction	−0.013	−0.193	0.061	0.955 (0.898–1.015)
Location	−0.004	−0.130	0.220	0.974 (0.913–1.040)
Mother's education level	0.006	0.122	0.248	1.023 (0.936–1.117)
Father's education level	0.008	0.154	0.145	0.905 (0.814–1.007)

*OR, odds ratio; CI, Confidence Interval; Coeff, coefficient; Std Coeff, standard coefficient*.

Tables 6, 7 show the differences in the scores of each instrument between the two patient subgroups (aged ≤24 vs. aged >24 months). In the ≤24 months group, there were significant differences in the HRQOL between patients with and without KMP in physical symptoms and physical and emotional functioning (*P* < 0.05). In the age >24 months group, the HRQOL for physical, emotional and social functioning were significantly different between patients with and without activity dysfunction (*P* = 0.000, *P* = 0.030, and *P* = 0.012, respectively).

**Table 6:** Analysis and score for each dimension of the PedsQL^TM^ 4.0 Infant Scales (age ≤24 months).

**PedsQL^**TM**^4.0 Infant Scales**	**Physical functioning (mean ±SD)**	**Physical symptom functioning (mean ±SD)**	**Emotional functioning (mean ±SD)**	**Social functioning (mean ±SD)**	**Cognitive functioning (mean ±SD)**
Normal group	92.39 ± 9.49	90.78 ± 7.84	90.44 ± 8.17	91.07 ± 10.69	88.12 ± 17.14
**Patient group**					
With KMP	67.43 ± 18.19	77.03 ± 16.45	62.44 ± 16.34	81.45 ± 15.58	77.40 ± 21.55
Without KMP	87.17 ± 11.34	86.90 ± 11.86	80.85 ± 14.46	97.44 ± 3.63	93.75 ± 9.93
*P*-value	0.000	0.048	0.000	0.000	0.001
Activity dysfunction	81.92 ± 16.62	85.89 ± 17.25	73.82 ± 18.19	92.85 ± 13.71	88.39 ± 20.32
Without activity dysfunction	72.86 ± 18.73	79.17 ± 14.60	68.27 ± 17.87	85.96 ± 14.60	82.26 ± 19.20
*P*-value	0.075	0.483	0.696	0.202	0.326
**Location**					
Extremity	71.26 ± 18.75	79.89 ± 14.30	68.47 ± 17.09	84.55 ± 16.10	81.00 ± 23.02
Trunk	77.45 ± 19.53	84.41 ± 12.54	73.04 ± 19.59	89.63 ± 12.24	87.81 ± 14.07
Head, face or neck	78.87 ± 16.79	78.39 ± 20.21	67.71 ± 17.97	90.63 ± 14.66	83.63 ± 19.63
*P*-value	0.786	0.925	0.382	0.587	0.979
**Mother's education level**					
Elementary education	68.06 ± 19.84	80.25 ± 14.41	62.50 ± 18.64	86.00 ± 18.72	85.14 ± 18.02
Secondary education	81.94 ± 13.19	86.11 ± 13.06	76.85 ± 15.18	89.72 ± 10.17	87.04 ± 16.20
Higher education	75.60 ± 18.99	79.78 ± 16.44	69.98 ± 18.09	87.79 ± 14.55	82.67 ± 21.022
*P*-value	0.459	0.572	0.097	0.965	0.393
**Father's education level**					
Elementary education	66.81 ± 16.44	82.00 ± 13.58	63.33 ± 18.40	83.50 ± 18.31	82.64 ± 17.46
Secondary education	78.47 ± 12.24	77.75 ± 12.93	70.42 ± 17.81	91.13 ± 9.56	87.15 ± 14.43
Higher education	76.84 ± 20.22	81.59 ± 16.91	71.47 ± 17.97	88.07 ± 14.68	83.26 ± 21.65
*P*-value	0.273	0.926	0.387	0.459	0.892

*SD, standard deviation*.

**Table 7:** Analysis and score of each dimension of the PedsQL^TM^ 4.0 Generic Core Scale (age >24 months).

**PedsQL^**TM**^4.0 Generic Core Scales**	**Physical functioning (mean ±SD)**	**Emotional functioning (mean ±SD)**	**Social functioning (mean ±SD)**	**School functioning (mean ±SD)**
Healthy children	89.06 ± 8.23	85.48 ± 13.47	89.88 ± 14.42	91.38 ± 9.19
**Patient group**				
With KMP	64.46 ± 22.03	65.31 ± 18.12	69.38 ± 18.79	65.24 ± 20.88
Without KMP	66.90 ± 24.33	73.41 ± 18.22	79.78 ± 21.74	71.49 ± 21.13
*P*-value	0.979	0.270	0.470	0.582
Activity dysfunction	50.35 ± 22.89	63.33 ± 18.86	64.72 ± 21.93	64.27 ± 25.38
Without activity dysfunction	79.84 ± 11.76	76.00 ± 16.11	85.55 ± 14.78	73.14 ± 15.23
*P*-value	0.000	0.030	0.012	0.091
**Location**				
Extremity	62.28 ± 22.75	74.64 ± 20.61	73.21 ± 22.50	67.44 ± 20.36
Trunk	69.59 ± 20.88	70.00 ± 16.04	77.00 ± 18.97	68.94 ± 17.60
Head, face or neck	65.28 ± 28.62	62.78 ± 18.05	76.11 ± 23.69	70.74 ± 27.13
*P*-value	0.999	0.882	0.519	0.341
**Mother's education level**				
Elementary education	65.97 ± 14.78	78.89 ± 16.91	78.33 ± 22.22	68.52 ± 13.88
Secondary education	76.39 ± 21.60	79.44 ± 13.57	85.56 ± 12.61	74.07 ± 18.52
Higher education	61.10 ± 26.00	61.75 ± 17.57	69.50 ± 22.12	65.96 ± 24.12
*P*-value	0.257	0.439	0.315	0.657
**Father's education level**				
Elementary education	55.68 ± 23.79	62.27 ± 14.03	70.91 ± 18.00	60.00 ± 19.20
Secondary education	71.18 ± 10.22	78.33 ± 18.54	77.22 ± 20.78	73.52 ± 9.84
Higher education	69.45 ± 26.18	70.56 ± 19.55	77.22 ± 23.28	71.33 ± 25.78
*P*-value	0.347	0.148	0.761	0.568

*SD, standard deviation*.

## Discussion

In the present study, we mainly focused on the physical conditions and psychological feelings of patients with KHE. We aimed to understand the influence of KHE on the daily life of children and their families, as well as their satisfaction with physiological, psychological and social adaptation functioning in patients and their families under the influence of disease. We also analyzed the HRQOL influencing factors to understand their real living conditions and internal feelings.

We found that patients with KHE had a lower HRQOL than those without. Patients aged >24 months showed differences in all scored entries, especially social functioning and cognitive functioning, compared with the normal control group. Children's self-consciousness and the development of psychological functioning gradually develop as they get older. Two-year-old children usually enter kindergarten and begin to develop social bonds with other children. However, because of their sickness-induced absence from school, they may feel inferior and lose confidence, ultimately resulting in a decline in performance. School social environment has a potential impact on HRQOL. Social support from classmates and teachers can promote the rehabilitation and long-term treatment adjustment of patients. Therefore, we encourage sick children to return to school and society as soon as possible when it is medically feasible. The disease duration of KHE may be long, which may affect patients' mental health and daily life. Therefore, additional efforts are needed to strengthen psychological support, reduce depression, and improve enthusiasm for learning in patients with KHE.

Parents of children with infantile hemangioma (a benign vascular tumor) often feel worried and anxious about their children's disease, especially parents of those with lesions on the face or other body parts that are not generally covered with clothes (15). However, in patients with KHE, we found that there was no significant difference in the HRQOL among patients with different lesion locations. This may be because KHE is a rare disease, and it is more difficult to treat than infantile hemangioma. These reasons may aggravate parents' anxiety and worry regardless of where the tumor is located. The prognosis of KHE was related to the tumor site, the degree of infiltration and the presence of KMP. Some patients without KMP will later develop KMP. Some patients with KMP will encounter further decreases in coagulation function. Patients without KMP may even experience a decreased range of motion. The presence of these complications suggests that the disease is progressing, and the morbidity rate will increase. As a consequence, parents may be concerned not only about the risk of disfigurement and the ridicule from other children but also about the child's health status itself. Therefore, it was not surprising that patients with KMP and activity dysfunction were associated with a relatively low HRQOL. Both factors affect patients' HRQOL.

One major concern of patients' parents is whether KHE can be effectively treated. Due to the lack of standard diagnosis and standard treatment, many patients with KHE do not receive appropriate treatment before referral (16). In general, the longer the disease progresses, the worse the HRQOL in children may be. Consistent with our previous study, we revealed that musculoskeletal complications occurred most commonly in older children (17). The destructive growth patterns associated with KHE and the infiltration of the muscles, connective tissues and joint structures can cause pain and functional limitations, all of which may affect a patient's abilities to perform routine daily activities. The difference between a patient and their peers can easily lead to the former experiencing negative social psychology, and patients with KHE may have difficulty establishing harmonious social relations. In addition, some children with effective initial treatment may later exhibit drug resistance, disease relapse or side effects. Going back and forth between hospitals and home greatly affects the physiological and psychological functioning of patients and parents, which may result in a decline in the HRQOL of children. We found that if KHE can be diagnosed at an early stage and in a timely manner, effective interventions can be applied (18). The incidence of complications and corresponding side effects of long-term treatment can be reduced. Moreover, psychosocial trauma can be prevented, and the long-term HRQOL of patients can be improved. If necessary, medical staff should provide necessary psychological intervention measures for patients at different ages.

When we evaluated the HRQOL in patients with KHE, we found that patients and their families had physical and psychological problems that affected their HRQOL. In this regard, treatment intervention should be carried out early. The improvement in the QOL can be used as a reference index to evaluate the therapeutic effect.

However, lesion location and the education level of the mother or father were not related to the HRQOL. Mothers are often the primary caregivers of children, and their emotional and psychological fluctuations can directly affect the psychological behavior of children. Although there was no statistical significance in the comparison of the educational experiences of mothers or fathers, it is interesting to note that the QOL scores of mothers with a primary education or higher were lower than those of mothers with only a secondary education. This may be due to the possibility that mothers with less than a primary education had insufficient knowledge of KHE, and it was difficult to obtain support and information related to the disease. Therefore, the cognitive function and psychological state of the child continue to deteriorate, leading to a decline in the HRQOL. However, mothers who obtain a higher education usually try to balance work and family and lack communication with their children, making it difficult for the children to receive appropriate nursing care and psychological care. Additionally, paying extra attention to a sick child consumes a mother's energy. The mother may be prone to excess anxiety, leading to a serious decline in the HRQOL.

Finally, we found that KMP and activity dysfunction were risk factors for a poor HRQOL in patients with KHE. For patients with these complications, good and targeted medical guidance and instructions can help them adjust their psychosocial and emotional conditions.

## Conclusions

The findings presented here suggest that KHE can influence the HRQOL in young patients and their parents. Patients with KHE and their parents generally have a poor HRQOL. Our results suggest that the combined use of the PedsQL™ 4.0 Infant Scales, FIM and GCS is sufficient to evaluate QOL in children with KHE. In addition, our study provides novel findings that KMP and activity dysfunction are risk factors for HRQOL and life satisfaction. We hope that future multicenter, prospective data from a large sample will be collected to support and extend these findings, with the aim of improving the HRQOL in patients with KHE.

## Data Availability Statement

The raw data supporting the conclusions of this article will be made available by the authors, without undue reservation.

## Ethics Statement

The studies involving human participants were reviewed and approved by the Ethics Committee of the West China Hospital of Sichuan University and the Ethics Committee of the Second West China Hospital of Sichuan University. Informed consent was obtained from the patients' parents. Written informed consent to participate in this study was provided by the participants' legal guardian/next of kin.

## Author Contributions

SD, KY, TQ, JZ, XZ, SC, LL, and YJ were involved in the initial conception and design of the study, data collection, and analysis of the data in this study. SD reviewed the literature and drafted the manuscript. LL and YJ reviewed the manuscript. All authors read and approved the final manuscript.

## Funding

This project was supported in part by the National Natural Science Foundation of China (81401606 and 81400862), Key Project in the Science and Technology Program of Sichuan Province (2019YFS0322), Science Foundation for the Excellent Youth Scholars of Sichuan University (2015SU04A15), and 1·3·5 project for disciplines of excellence-Clinical Research Incubation Project of West China Hospital of Sichuan University (2019HXFH056).

## Conflict of Interest

The authors declare that the research was conducted in the absence of any commercial or financial relationships that could be construed as a potential conflict of interest.

## Publisher's Note

All claims expressed in this article are solely those of the authors and do not necessarily represent those of their affiliated organizations, or those of the publisher, the editors and the reviewers. Any product that may be evaluated in this article, or claim that may be made by its manufacturer, is not guaranteed or endorsed by the publisher.

## Abbreviations

KHE: Kaposiform hemangioendothelioma
HRQOL: health-related quality of life
KMP: Kasabach-Merritt phenomenon
QOL: quality of life
PedsQL™ 4.0: Pediatric Quality of Life Inventory Version 4.0
FIF: Family Information Form
FIM: Family Impact Module
GCS: Generic Core Scales
SD: standard deviation.
